# Optimizing Machine Learning with SSA and PSO for Anchor Bolt–Grout Bond Strength Prediction

**DOI:** 10.3390/ma19050906

**Published:** 2026-02-27

**Authors:** Detan Liu, Chenglin Liu, Hongwei Zhang, Meng Cui, Chuankai He, Junjie Wang

**Affiliations:** 1 Datang Hydropower Science & Technology Research Institute Co., Ltd., Chengdu 610083, China; ldthhu@126.com (D.L.); chenyanyan2026@126.com (M.C.); kaichuanhe@126.com (C.H.); 2 College of Civil and Transportation Engineering, Hohai University, Nanjing 210098, China; 3 School of Materials Science and Engineering, Chongqing Jiaotong University, Chongqing 400074, China; 4 College of Water Conservancy and Hydropower Engineering, Hohai University, Nanjing, 210098, China; zhanghongwei@hhu.edu.cn

**Keywords:** machine learning, interface bond strength, corroded anchor bolts, SHAP analysis, sparrow search algorithm, particle swarm optimization algorithm

## Abstract

The bond strength (*τ*) of the interface between the anchor bolt and grouting body (or rebar–concrete) is a key indicator used to evaluate the bearing capacity of anchorage engineering. And when rebars are subject to corrosion, *τ* also serves as an important durability metric. However, traditional experimental measurement of *τ* is complex, time-consuming and labor-intensive. In this study, based on pullout test data from 429 rebar–concrete specimens, we develop a machine learning method to construct a prediction model with strong generalization ability. Fundamental features—including specimen geometry, dimensions, material strengths, and corrosion rate—are used as inputs. The Sparrow Search Algorithm (SSA) and Particle Swarm Optimization (PSO) are used to fine-tune the hyperparameters of three machine learning models which are Random Forest (RF), Least Squares Boosting (LSBoost), and Generalized Additive Model (GAM). We perform a comparative error analysis of each model and benchmark them against three empirical formulas for *τ*. The unoptimized models exhibit low predictive accuracy and clear overfitting. After optimization using SSA and PSO algorithms, the prediction accuracy and overfitting issues are significantly improved, with the PSO-LSBoost model achieving the best performance (*R*^2^ = 0.93). The PSO-LSBoost model’s prediction accuracy for *τ* far exceeds that of the three empirical formulas. SHAP analysis reveals that the corrosion rate (*C*_w_) contributes most to *τ*, while the rebar type (*ST*) contributes least. This work introduces a novel, efficient approach for predicting anchorage bond strength and assessing bolt durability, thereby enhancing the reliability of anchorage structures.

## 1. Introduction

Anchoring technology is an economical and effective means of rock mass reinforcement. Due to its high stiffness and bearing capacity, it has been widely applied in fields such as railways, highways, water conservancy, and mining. When anchor bolts are used in complex environments, their durability can rapidly decrease due to factors such as wet-dry cycles, corrosive media (sulfates, chlorides, etc.), and extreme weather conditions. Statistics show that the number of deaths caused by anchorage structure failures accounts for 17% of deaths from natural disasters each year [1]. For example, in both the UK and China, several anchorage structures have failed; investigations reveal that corrosion of anchor bolts was responsible for these failures, resulting in reduced bearing capacity [2,3]. The bearing capacity of anchorage structures mainly comes from the bond strength between the anchor bolt–grouting body (or rebar–concrete) interface [4,5]. Therefore, it is crucial to identify the factors influencing the bond strength and degradation law of corroded anchor bolt–grouting body (or rebar–concrete) interfaces, to guide targeted improvement of the structure’s durability during design.

Numerous scholars have investigated the bond behavior of corroded reinforcing steel embedded in concrete and have proposed several models for estimating bond strength. These models are predominantly based on conventional regression analyses and are typically validated using limited experimental datasets. For instance, Bhargava et al. [6], drawing on earlier experimental results, developed an empirical model describing the degradation of bond strength induced by steel corrosion. However, this model was derived from flexural tests on beams and therefore exhibits discrepancies when applied to pull-out tests of anchored bars. Yang Xiaoming et al. [7] suggested that the bond strength between corroded reinforcement and concrete can be expressed as the bond strength of uncorroded reinforcement multiplied by a degradation coefficient. Based on regression analysis, they proposed a predictive model that considers only the corrosion rate. Owing to the inclusion of a single influencing parameter, this model lacks general applicability. Chen Zhaohui et al. [8] accounted for four factors—corrosion product thickness, relative concrete cover thickness, anchorage length, and concrete compressive strength—and proposed an empirical model for predicting the bond strength of corroded anchor bars. Nevertheless, the model is applicable only to anchor bars with corrosion rates below 5%, rendering it inherently case-specific. In addition, numerous empirical models for the bond strength of corroded reinforcement have been proposed by other researchers [9]. Similar to the aforementioned models, their predictive reliability remains high only under specific conditions.

In reality, the bond performance of reinforced concrete is governed by multiple interacting factors, and the underlying mechanisms are highly complex. Concrete compressive strength is widely recognized as one of the most critical parameters influencing bond behavior [10,11]. Furthermore, factors such as the ratio of concrete cover thickness to bar diameter [10,12,13], the degree of transverse confinement [14], bond length [15], and the geometric characteristics of the reinforcement cross-section [16] also exert significant effects on bond performance. For example, Mohamed and Clark [17] evaluated the influences of reinforcement type, concrete cover thickness, and transverse reinforcement on bond strength. Ahmad et al. [18,19], focusing on lightweight aggregate concrete, developed bond–slip models and bond strength formulations, highlighting the roles of development length, cover thickness, and reinforcement type. Boonmee and Rodsin [20] reported that bond strength is more strongly dependent on concrete compressive strength than on bar diameter. Bicakci et al. [21,22] demonstrated that increasing coarse aggregate content enhances both bond strength and residual bond stress in lightweight aggregate concrete, while simultaneously improving its compressive and tensile properties. Conventional regression-based approaches often struggle to achieve satisfactory predictive accuracy when addressing such multivariate problems [23,24,25].

Predicting the interfacial bond strength (*τ*) of anchors (reinforced concrete) using traditional pull-out tests combined with regression analysis is not only labor- and cost-intensive but also makes it difficult to establish models that are both broadly applicable and capable of simultaneously accounting for multiple influencing variables. Machine learning techniques offer a fundamentally new avenue for predicting *τ*. In recent years, with the rapid development of artificial intelligence, machine learning has attracted increasing attention across a wide range of disciplines [26]. For example, Ling et al. [27] employed the POA algorithm to optimize the hyperparameters of four conventional machine learning models—RFR, MLP, GAM, and LSBoost—for predicting the three-point bending fracture load of asphalt concrete. Comparative analysis with fracture criteria indicated that the POA-LSBoost model achieved superior predictive accuracy and reliability. Naranjo-Pérez et al. [28] integrated harmony search, the active set method, artificial neural networks, and principal component analysis to develop a cooperative algorithm for finite element model updating, which substantially reduced computational time and improved the robustness of selecting optimal updated models. In the field of anchorage technology, Suenaga et al. [29] investigated the predictive performance of three machine learning models—Random Forest (RF), XGBoost, and LightGBM—for anchor shear capacity. Hoang et al. [30] considered six potential feature variables and employed a metaheuristically optimized least-squares support vector regression model to predict the bond strength of reinforced concrete; however, the coefficient of determination (*R*^2^) reached only 0.84, indicating limited predictive accuracy. Mohsen Ebrahimzadeh et al. [31] applied Decision Tree (DT), Random Forest (RF), Gradient Boosting (GB), and XGBoost (XGB) models to predict reinforced concrete bond strength and optimized these models using grid search. Although the optimized XGB model achieved a relatively high *R*^2^ value of 0.90, the study focused on flexural test data from reinforced concrete beams, which differ from pull-out test conditions. T. Liu et al. [32] proposed a bond strength prediction framework for reinforcement–mechanically stabilized gravel concrete (MGC) by combining machine learning algorithms with SHapley Additive exPlanations (SHAP) and partial dependence plots (PDP). Their results showed that the Random Forest model attained an *R^2^* of 0.95; however, the applicability of the model is limited to MGC–reinforcement systems. Yixun Yu et al. [33] compiled 281 pull-out test datasets of fiber-reinforced polymer (FRP) bars embedded in fiber-reinforced concrete (FRC) to train and evaluate various machine learning models, including support vector machines (SVM), decision trees (DT), random forests (RF), gradient boosting decision trees (GBDT), AdaBoost, and XGBoost. Through comprehensive comparison with five existing empirical equations, they demonstrated the superior predictive accuracy of machine learning models. Nevertheless, the scope of this study is similarly restricted to FRP bars and FRC.

Artificial neural networks (ANNs) are among the most widely adopted machine learning regression models. Concha et al. [34] considered four factors—compressive strength, anchorage length, the ratio of concrete cover thickness to bar diameter, and ultrasonic pulse velocity—and employed an ANN model to analyze 108 laboratory pull-out test results, achieving predictions closely aligned with experimental observations. Wang et al. [35] combined ANNs with genetic algorithms (GA) and particle swarm optimization (PSO) to train and test a dataset comprising 191 pull-out tests. The developed PSO-ANN model exhibited excellent predictive performance, with *R*^2^ values of 0.92 and 0.93 for the training and testing datasets, respectively, significantly outperforming conventional ANN models in bond strength prediction. However, these models are constrained by relatively small sample sizes or limited feature variables. In addition, Golafshani et al. [36] applied ANN methods to predict the bond behavior of spliced reinforcement in concrete, Hwang et al. [37] used ANNs to estimate the bond performance of tension lap splices, and Haddad et al. [38] employed ANNs to predict the bond strength of fiber-reinforced polymer–concrete interfaces. Despite their widespread application in concrete engineering, ANNs suffer from inherent limitations in search strategies. During network training, inappropriate initialization of weights and biases may lead ANNs to converge to local optima rather than the global optimum [39]. This occurs because ANN models rely on gradient descent algorithms, which proceed along the direction of steepest descent; consequently, if the initial solution is far from the global minimum, the optimization process can become trapped in local minima. Moreover, poorly initialized ANNs are susceptible to overfitting, exhibiting strong performance during training but poor generalization during testing.

In summary, existing models are characterized by either limited predictive accuracy, restricted applicability, or insufficient sample sizes. To address these limitations and enhance model generalizability, this study compiles an extensive dataset comprising 429 pull-out test results collected from 20 published studies and incorporates a broader range of feature variables. The predictive performances of three machine learning models—Random Forest (RF), Least Squares Boosting (LSBoost), and Generalized Additive Model (GAM)—for *τ* are systematically compared. Particle Swarm Optimization (PSO) and the Sparrow Search Algorithm (SSA) are employed to optimize model hyperparameters, thereby improving predictive accuracy and mitigating overfitting. Finally, the proposed models are benchmarked against existing machine learning approaches and empirical formulations from the literature, and SHAP analysis is conducted to interpret the predicted *τ* values. This work overcomes the high labor intensity and long duration associated with traditional experimental methods, enabling efficient and accurate prediction of the bond strength of corroded anchor bars, and provides valuable insights for future durability studies in anchorage engineering.

## 2. Data Collection

Based on an extensive review of the literature, this study compiles 20 experimental investigations published between 2000 and 2024 that focus on the bond strength of corroded anchor bars [40,41,42,43,44,45,46,47,48,49,50,51,52,53,54,55,56,57,58,59], yielding a total of 429 datasets. The Appendix A provides detailed information on the source references and experimental conditions for all collected data. Table 1 presents the statistical descriptions of all variables. As shown in Table 1, seven key input variables are considered in this study: bar diameter (*D*_s_), anchorage length (*L*_b_), concrete compressive strength (*f*_c_), minimum concrete cover thickness (*C*_min_), reinforcement type (*ST*), presence of transverse reinforcement (*Stirrup*), and corrosion rate (*C*_w_). The output variable is the bond strength (*τ*). It should be noted that reinforcement strength is not included as an input variable in this study. This omission is justified by the fact that, for all 429 pull-out test specimens, the stresses developed in the reinforcement during testing were far below the corresponding yield strengths. Consequently, the influence of reinforcement strength on the experimental outcomes can be reasonably neglected.

The mean reflects the average level of the data and is sensitive to extreme values, whereas the median represents the central tendency and is robust to outliers. The standard deviation quantifies the dispersion of data points around the mean. Together, these three statistics provide a concise characterization of the underlying data distributions. Notably, substantial discrepancies between the mean and median are observed for *C*_w_ and *τ,* indicating that most data points are concentrated within a relatively narrow range, while a limited number of extreme values elevate the mean. This suggests a non-normal distribution for these variables. The relatively large standard deviations of *L*_b_ and *C*_min_ indicate a wide spread and pronounced variability among individual observations. In contrast, the remaining variables exhibit small differences between their means and medians, along with relatively low standard deviations, implying approximately symmetric and concentrated distributions.

**Figure 1:**
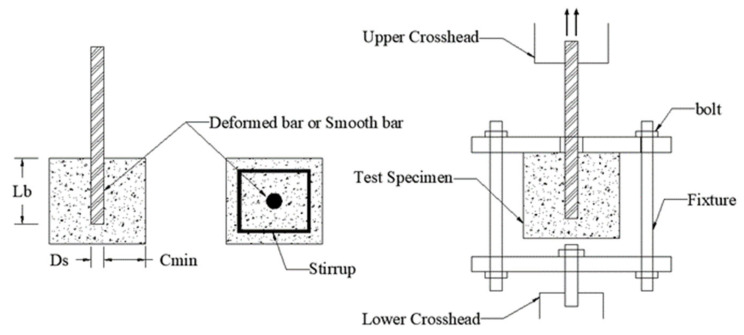
Schematic diagram of typical specimen and loading configuration.

**Figure 2:**
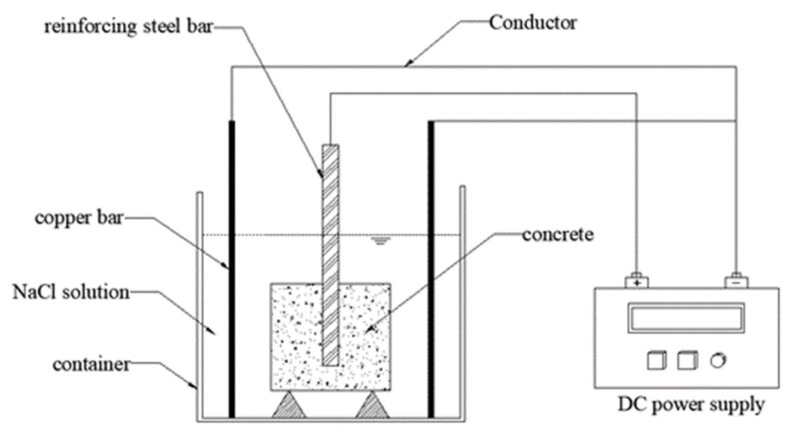
Schematic diagram of typical accelerated current corrosion.

All feature parameters were directly measured or calculated from the experiments. The *f*_c_ values represent the 28-day compressive strength of concrete, *ST* has two values (1 represents deformed rebar, 2 represents plain round rebar), and *Stirrup* also has two values (1 represents the presence of stirrups, 2 represents no stirrups). *τ* was obtained through axial pullout tests, with a typical specimen structure and loading method, as shown in Figure 1. The calculation formula for *τ* is given by Equation (1), where the bond strength value represents the average stress at the bond interface. Although in practical engineering, the bond stress along the anchor bolt interface is unevenly distributed, in the experiments, however, the anchorage length was relatively short, and under the ultimate load, which means it can be approximated as uniformly distributed along the anchor bolt length [60]. The corroded specimens were prepared using the post-casting corrosion method, which is more representative of actual engineering situations. Figure 2 shows a typical schematic of the current accelerated corrosion method. Equation (2) provides the expression for calculating *C*_w_, which represents the average corrosion rate of each rebar. To ensure consistency beyond the seven independent variables, study [42] used only post-corrosion testing, while study [53] included data exclusively from experiments conducted at ambient temperature. Overall, the dataset is extensive, compiled from diverse studies, and encompasses multiple variables, making it reasonably representative.

(1) $$\tau =\frac{P_{max}}{\pi \times D_{s}\times L_{b}}\;$$

(2) $$C_{w}=\frac{m_{0}-m_{f}}{m_{0}}\times 100\%\;$$

where *P*_max_ is the failure load in the pullout test (N), *D*_s_ is the rebar diameter (mm), and *L*_b_ is the anchorage length (mm). *m*_0_ is the initial mass of the rebar before corrosion, and *m_f_* is the remaining mass of the rebar after cleaning off the corrosion products.

**Figure 3:**
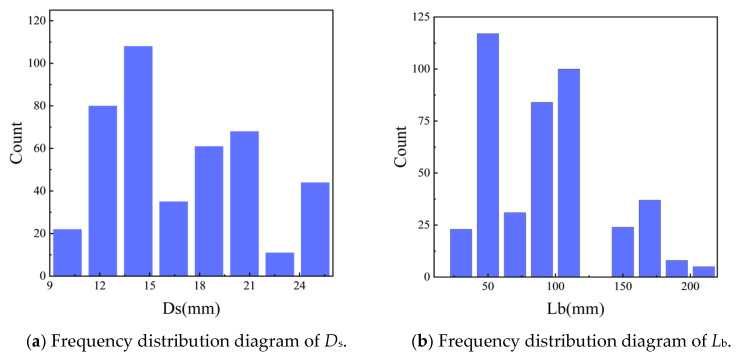

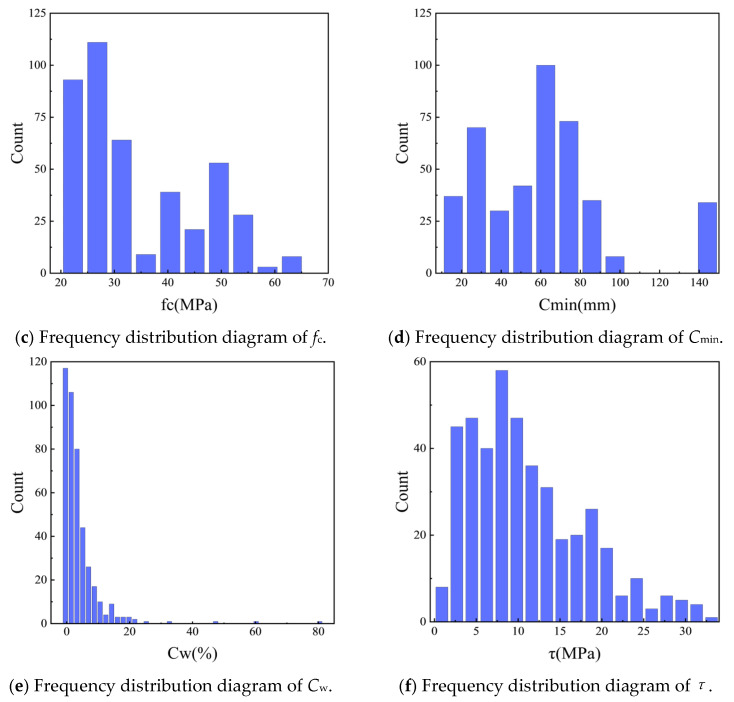
Frequency distribution of partial characteristic factors versus *τ*.

Figure 3 presents the frequency distributions of anchor bond strength and selected feature parameters for the 429 collected datasets, providing a more intuitive representation of the statistical characteristics of the variables and serving as a reference for the subsequent correlation analysis. As shown, the anchor bar diameter (*D*_s_) values are primarily concentrated within the range of 10–25 mm and exhibit an approximately normal and relatively uniform distribution. The datasets for anchorage length (*L*_b_) and concrete cover thickness (*C*_min_) also approximately follow normal distributions; however, they are unevenly distributed, with missing values in the mid-range intervals. The frequency of concrete compressive strength (*f*_c_) decreases with increasing *f*_c_. The corrosion rate of anchor bars (*C*_w_) is largely concentrated below 0.2, although a small number of extreme values are present. From a practical engineering perspective, excessively high corrosion rates are of limited relevance; once the corrosion rate exceeds approximately 20%, the load-carrying capacity of anchor bars deteriorates rapidly and may even be completely lost. Steel type (*ST*) and the presence of stirrups (*Stirrup*) exhibit bimodal distributions, as each variable assumes only two discrete values.

### Selection of Characteristic Parameters

In machine learning, correlation analysis of feature variables constitutes a critical step in data preprocessing and feature engineering. Correlation analysis not only helps identify features that are strongly associated with the target variable, but also enables the assessment of inter-feature independence, thereby preventing multicollinearity. This process facilitates the elimination of irrelevant or redundant features, reduces model complexity, and mitigates the risk of overfitting. Correlation is commonly quantified using the Pearson or Spearman correlation coefficients. The Pearson correlation coefficient is appropriate for data that approximately follow a normal distribution and exhibit linear relationships, but it is sensitive to outliers. This sensitivity arises because Pearson correlation measures linear dependence; as expressed in Equation (3), it effectively seeks the best-fitting straight line that maximizes the closeness of data points to that line. Consequently, when the true relationship between variables is nonlinear, Pearson correlation may yield a low coefficient even in the presence of a clear underlying pattern. In contrast, the Spearman correlation coefficient is well suited for data with unknown distributions, the presence of outliers, or nonlinear relationships, and thus has a broader range of applicability. As defined in Equation (4), Spearman correlation assesses monotonic dependence: as long as two variables exhibit consistent directional trends (e.g., one tends to increase as the other increases, though not necessarily at a constant rate), the relationship can be detected. It is therefore effective for any monotonic increasing or decreasing function, including linear, exponential, and logarithmic relationships.

(3) $$Pearson’s=\frac{\sum_{i=1}^{n}\left(Y_{i}-\bar{Y})(X-\bar{X}\right)}{\sqrt{\sum_{i=1}^{n}{\left(Y_{i}-\bar{Y}\right)}^{2}}\sqrt{\sum_{i=1}^{n}{\left(X_{i}-\bar{X}\right)}^{2}}}$$

(4) $$Spearman’s=\frac{\sum_{i=1}^{n}\left(R_{i}-\bar{R})(Q_{i}-\bar{Q}\right)}{\sqrt{\sum_{i=1}^{n}{\left(R_{i}-\bar{R}\right)}^{2}}\sqrt{\sum_{i=1}^{n}{\left(Q_{i}-\bar{Q}\right)}^{2}}}$$

where $\bar{Y}$, $\bar{X}$: the mean values of *Y* and *X*; *R*_i_, *Q*_i_: the ranks of *Y* and *X*; and $\bar{R}$, $\bar{Q}$: the mean ranks of *R*_i_ and *Q*_i_.

**Figure 4:**
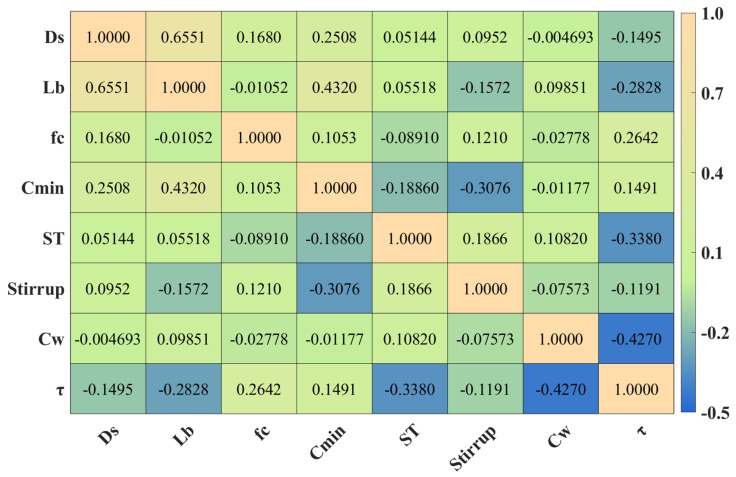
Spearman correlation matrix analysis.

In this study, several feature variables exhibit non-normal distributions (e.g., *f*_c_, *ST*, and *Stirrup*), and partial data gaps are present (e.g., missing *L*_b_ values in the range of 100–150 mm and *C*_min_ values in the range of 100–140 mm). In addition, the corrosion rate (*C*_w_) contains extreme outliers. Moreover, the relationships among variables are not necessarily linear. Under these conditions, the Spearman correlation coefficient is more appropriate for correlation analysis. Figure 4 presents the Spearman correlation matrix, from which it can be observed that *C*_w_, *ST*, and *f*_c_ exhibit relatively strong correlations with the target variable *τ*, in agreement with established engineering understanding. The corrosion rate *C*_w_ serves as an overall indicator of the durability of corroded anchor bars. *A*_s_ corrosion severity increases, bond strength *τ* decreases; consequently, *C*_w_ is negatively correlated with *τ*. Deformed reinforcement enhances mechanical interlocking between the steel bar and the surrounding concrete, thereby increasing anchor bond strength. The concrete compressive strength *f*_c_ reflects the load-bearing capacity of the concrete matrix; higher strength grades reduce the likelihood of anchorage failure and lead to increased bond strength. It is also evident that *D*_s_ and *L*_b_ are strongly correlated, indicating that these two variables are not independent. In fact, as indicated by the bond strength formulation in Equation (1), the predicted bond strength *τ* is defined as the ratio of the peak load to the interfacial contact area. Accordingly, *D*_s_ and *L*_b_ can be combined into a single feature representing the bond area, denoted as *A*_s_, which is expressed as Equation (5):

(5) $$A_{s}=\pi D_{s}L_{b}$$

When the correlation between a feature and the target variable is very weak, the decision to retain or remove that feature should be made cautiously. Although both *C*_min_ and *Stirrup* show low correlations with *τ*, all features exhibit correlation coefficients with *τ* below 0.427, indicating that *τ* does not have any strongly correlated single predictor. Therefore, it is inappropriate to remove both features simultaneously. Further analysis reveals that *C*_min_ shows relatively strong linear correlations with *L*_b_, *D*_s_, and *Stirrup*, suggesting that *C*_min_ is not an independent variable. Consequently, *C*_min_ is excluded from further analysis. Based on the comprehensive correlation assessment, five key feature parameters are ultimately retained as model inputs: *A*_s_, *f*_c_, *ST*, *Stirrup*, and *C*_w_.

## 3. ML Models

This study employs three different machine learning algorithms: Random Forest (RF), Least Squares Boosting (LSBoost), and Generalized Additive Model (GAM).

Random Forest (RF) is an ensemble learning algorithm with robust predictive performance and is widely used across machine learning applications. Proposed by Leo Breiman and Adele Cutler in 1995, RF improves the model’s accuracy and robustness by constructing multiple decision trees and combining their predictions. The Random Forest algorithm is founded on two fundamental principles: generating multiple bootstrap samples from the training data and training a separate decision tree on each sample; and at each split in a tree, randomly selecting a subset of features to determine the optimal partition. This design reduces the correlation between trees and enhances the diversity of the model. And the final prediction is made by averaging the predictions from all the decision trees. Least Squares Boosting (LSBoost) is an ensemble learning regression model based on the Boosting framework, which achieves high-accuracy predictions by iteratively optimizing the weighted combination of weak learners. The core principle combines the loss function of Least Squares Support Vector Machine (LSSVM) with the gradient boosting mechanism. The model first initializes a base learner (e.g., linear regression or decision tree), and in each iteration, it calculates the residuals of the current model’s predictions. Sample weights are adjusted based on the residuals, prioritizing training the samples with larger errors to generate new weak learners. The final prediction is obtained by integrating the outputs of all weak learners through a weighted averaging strategy. LSBoost is capable of handling both linear and nonlinear relationships in data, managing high-dimensional feature interactions, and also performing well in small sample scenarios. Generalized Additive Model (GAM) is a machine learning regression method that combines the interpretability of linear models with the nonlinear modeling capability, making it especially suitable for capturing complex nonlinear relationships between variables. The key concept of the GAM is to extend traditional linear regression by replacing its linear predictor terms with smooth functions—such as splines or kernel functions—thereby allowing each predictor to produce a flexible, potentially nonlinear effect on the response variable.

### 3.1. PSO and SSA Optimization Algorithms

Traditional models may suffer from issues such as inadequate prediction accuracy and overfitting risks when handling complex data with a large number of features. Particle Swarm Optimization (PSO) is a global optimization algorithm based on swarm intelligence, proposed by Kennedy J. et al. [61] in 1995, inspired by the foraging behavior of bird flocks. As a representative of metaheuristic algorithms, PSO has shown significant advantages in solving complex optimization problems due to its simple implementation, fast convergence speed, and strong parallelism. For example, in hyperparameter tuning for neural networks, PSO can avoid the vanishing gradient problem; in combinatorial optimization, its global search capability outperforms the performance of Genetic Algorithms (GA) in certain scenarios [62,63]. In the PSO algorithm, each particle represents a candidate solution in the solution space, and its position and velocity are updated during the iteration process. The position and velocity of the i-th particle in an n-dimensional space are denoted as *x*_i_ = (*x*_i1_, *x*_i2_, …, *x*_in_) and *v*_i_ = (*v*_i1_, *v*_i2_, …, *v*_i3_). The particle swarm adjusts its movement by tracking both the individual’s best historical position P and the global best position g. The velocity and position update formulas are given by Equations (6) and (7) [64]:

(6) $$v_{ij}^{t+1}=\omega^{t}v_{ij}^{t}+c_{1}r_{1}^{t}\left(P^{{best}_{i}}-x_{ij}^{t}\right)+c_{2}r_{2}^{t}\left(g^{best}-x_{ij}^{t}\right)$$

(7) $$x_{ij}^{t+1}=x_{ij}^{t}+v_{ij}^{t+1}$$

where *i* = 1, 2, …, *p*, *j* = 1, 2, …, *n*, *t* = 1, 2, …, *T*, *c*_1_ and *c*_2_ are the learning factors, *r*_1_ and *r*_2_ are random numbers, *P^besti^* is the best historical position of particle *i*, *g^best^* is the global best position of the swarm, and *ω^t^* is the inertia weight at time step *t*.

**Figure 5:**
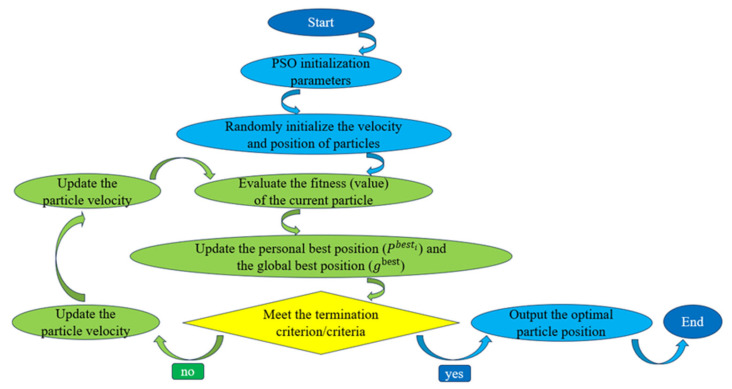
Flowchart of the PSO algorithm.

The performance of particle swarm optimization (PSO) is jointly governed by several key parameters, whose configuration directly affects the convergence rate, solution accuracy, and computational efficiency of the algorithm [65]. The principal parameters can be summarized as follows:

(1) Population size (*m*). The population size is typically recommended to be set between 20 and 50. A larger population enhances the global exploration capability of the algorithm but substantially increases computational cost, whereas an excessively small population may lead to insufficient diversity and a higher likelihood of premature convergence to local optima.

(2) Particle dimensionality (*D*). The particle dimensionality is determined by the dimensionality of the search space associated with the optimization problem [66,67,68,69,70]. In this study, for Random Forest optimization, two hyperparameters—the number of decision trees and the maximum number of features—are tuned (*D* = 2). For Least Squares Boosting, three parameters are optimized, namely the number of boosting iterations, the learning rate, and the complexity of the base learner (*D* = 3). For the Generalized Additive Model, the primary focus is on optimizing the regularization strength parameter (*D* = 1).

(3) Velocity and position bounds. The velocity range determines the particle step size during the search process. An excessively large range may cause particles to overshoot promising regions, whereas an overly small range can weaken global exploration capability. The position bounds define the feasible solution space; appropriately setting these bounds enables a balance between search efficiency and solution quality.

(4) Inertia weight (*ω*). The inertia weight controls the extent to which particles retain their current velocities, thereby mediating the trade-off between global exploration and local exploitation. Larger values of ω favor broad exploration of the search space, whereas smaller values encourage fine-grained searches in the vicinity of the current solution.

(5) Acceleration coefficients (*c*_1_, *c*_2_). These coefficients represent the relative influence of the particle’s personal best position and the global best position, respectively. Excessively large acceleration coefficients may induce oscillatory particle trajectories and cause particles to overshoot the optimal solution, while overly small values reduce learning efficiency, slow convergence, and increase the risk of entrapment in local optima.

(6) Maximum number of iterations (*T*_max_). This termination criterion should be determined according to problem complexity and available computational resources. An insufficient number of iterations may prevent convergence and yield highly dispersed solutions, whereas excessive iterations may marginally improve accuracy at the cost of unnecessary computational overhead. A schematic illustration of the PSO algorithm is shown in Figure 5.

The Sparrow Search Algorithm (SSA) was proposed by Xue, J. [71] in 2020, inspired by the foraging behavior and anti-predator strategies of sparrow populations. During the foraging process, sparrow groups exhibit a cooperative wisdom: some sparrows (the discoverers) are responsible for exploring food sources, while others (the followers) compete to acquire resources. Additionally, certain sparrows act as sentinels to avoid predation. By simulating this behavioral mechanism, SSA achieves an efficient balance between global exploration and local exploitation, significantly enhancing optimization performance. Due to its simple structure, fewer parameters, and fast convergence speed, SSA has demonstrated faster dynamic response and higher precision than PSO in some areas. In SSA, the sparrow population is divided into three roles: Producers are responsible for global search and guide the population towards the optimal region, Scroungers follow the discoverers and compete for local development, and Watchers randomly monitor the environment to prevent the population from getting trapped in local optima. The position update formulas are given by Equations (8)–(10) [72]:

(8) $$\mathrm{Producer:}\;x_{ij}^{t+1}=\{\begin{array}{c} x_{ij}^{t}\cdot EXP\left(-\frac{i}{\alpha \cdot Maxitem}\right),R_{2}<ST,\\ x_{ij}^{t}+Q\cdot L,\;\;R_{2}\geq ST.\; \end{array}$$

**Figure 6:**
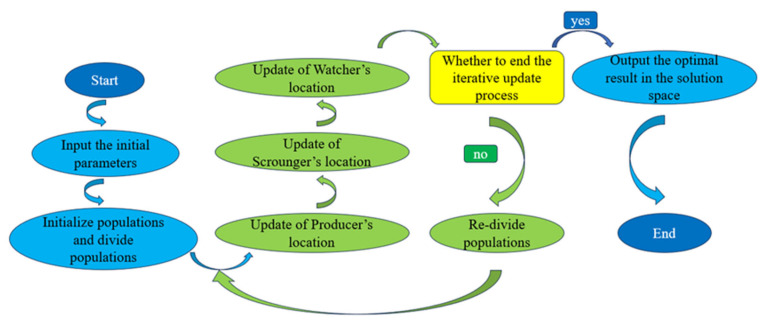
Flowchart of the SSA.

In this context, *x_ij_^t^*^+1^ represents the current position of the *i*-th sparrow at the *j*-th dimension at iteration *t*; *M*_axitem_ is the maximum number of iterations; *t* is the current iteration; *α* is a uniform random number drawn from the interval (0, 1); *Q* follows a standard normal distribution and is a random number; *L* is a 1 *× d* matrix with all elements equal to 1; *R*^2^ is a random number drawn from the interval (0, 1), and *ST* is a scaling factor in the range (0.5, 1).

(9) $$\mathrm{Scrounger:}\;x_{ij}^{t+1}=\{\begin{array}{c} Q\cdot EXP\left(\frac{X_{worst}-X_{ij}^{t}}{i^{2}}\right),\;i>\frac{n}{2},\\ x_{bj}^{t+1}+\left|x_{ij}^{t}-x_{bj}^{t+1}\right|\cdot A^{+}\cdot L,\;else.\; \end{array}$$

where *X_worst_* represents the global worst position, *x_bj_* denotes the global best position in the *j*-th dimension at the *t* + 1-th iteration, A is a 1 *× d* matrix with elements randomly composed of 1 and −1, A^+^ = A^T^(AA^T^)^−1^.

(10) $$\mathrm{Watcher:}\;x_{ij}^{t+1}=\{\begin{array}{c} x_{ij}^{t}+\beta \left|x_{ij}^{t}-x_{bj}^{t}\right|,\;f_{i}\neq f_{g},\\ x_{bj}^{t+1}+K\frac{x_{ij}^{t}-x_{worst,j}^{t}}{\left(f_{i}-f_{worst}\right)+\varepsilon },\;f_{i}=f_{g}.\; \end{array}$$

where *K* is in the range (0, 1), and it is a random number, *ε* is an infinitesimally small number, and *f*_i_, *f*_g_, *f*_worst_ represent the current fitness, global best fitness, and global worst fitness values, respectively. Figure 6 is a schematic diagram of the SSA [73].

### 3.2. Error Evaluation Criteria

In this study, the model’s prediction performance is compared using the coefficient of determination (*R*^2^), mean absolute error (*MAE*), mean relative error (*MRE*), and root mean square error (*RMSE*) [74], with their expressions given by Equations (11)–(14). *R*^2^ quantifies the proportion of variance in the experimental values explained by the model’s predictions, thereby indicating the accuracy of those predictions. *MAE* measures the absolute difference between the predicted and experimental values, while *MRE* quantifies the relative difference between the model’s predictions and the experimental values. *RMSE* is another important indicator commonly used to assess the model’s prediction accuracy. All three metrics reflect the model’s prediction accuracy and error level to some extent, and smaller values indicate higher model accuracy and lower errors.

(11) $$R^{2}=1-\frac{\sum_{i=1}^{n}{\left(y_{i}-{\hat{y}}_{i}\right)}^{2}}{\sum_{i=1}^{n}{\left(y_{i}-\bar{y}\right)}^{2}}$$

(12) $$MAE=\frac{\sum_{i=1}^{n}\left|y_{i}-{\hat{y}}_{i}\right|}{n}$$

(13) $$MRE=\frac{\sum_{i=1}^{n}\frac{\left|y_{i}-{\hat{y}}_{i}\right|}{y_{i}}}{n}$$

(14) $$RMSE=\sqrt{\frac{\sum_{i=1}^{n}{\left(y_{i}-{\hat{y}}_{i}\right)}^{2}}{n}}$$

## 4. Results and Discussion

### 4.1. Model Prediction Results

**Figure 7:**
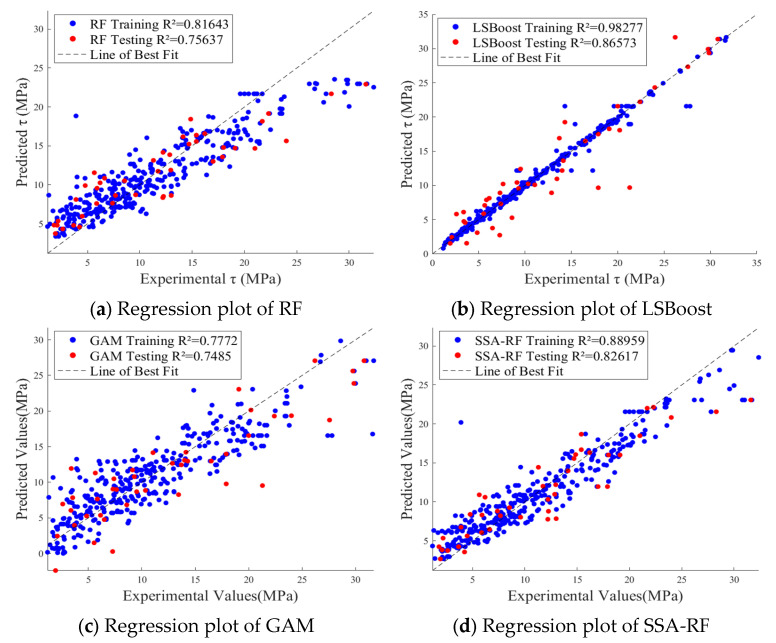

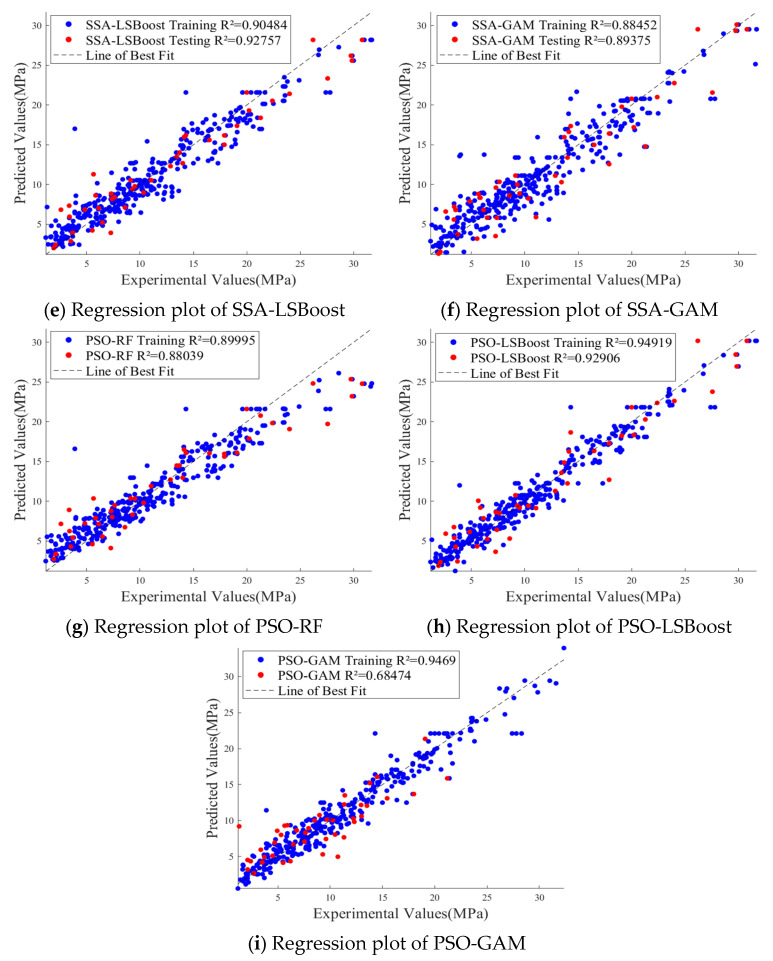
Regression plots of different models.

**Figure 8:**
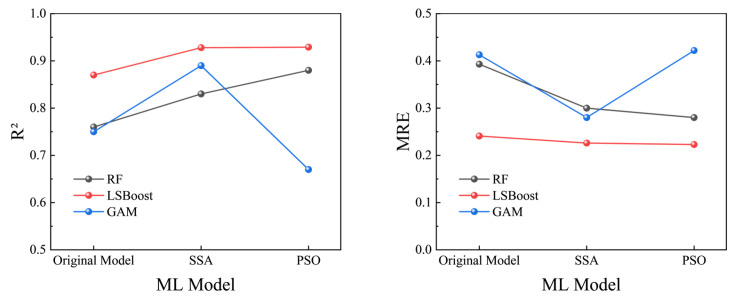
Error comparison of different algorithms.

**Figure 9:**
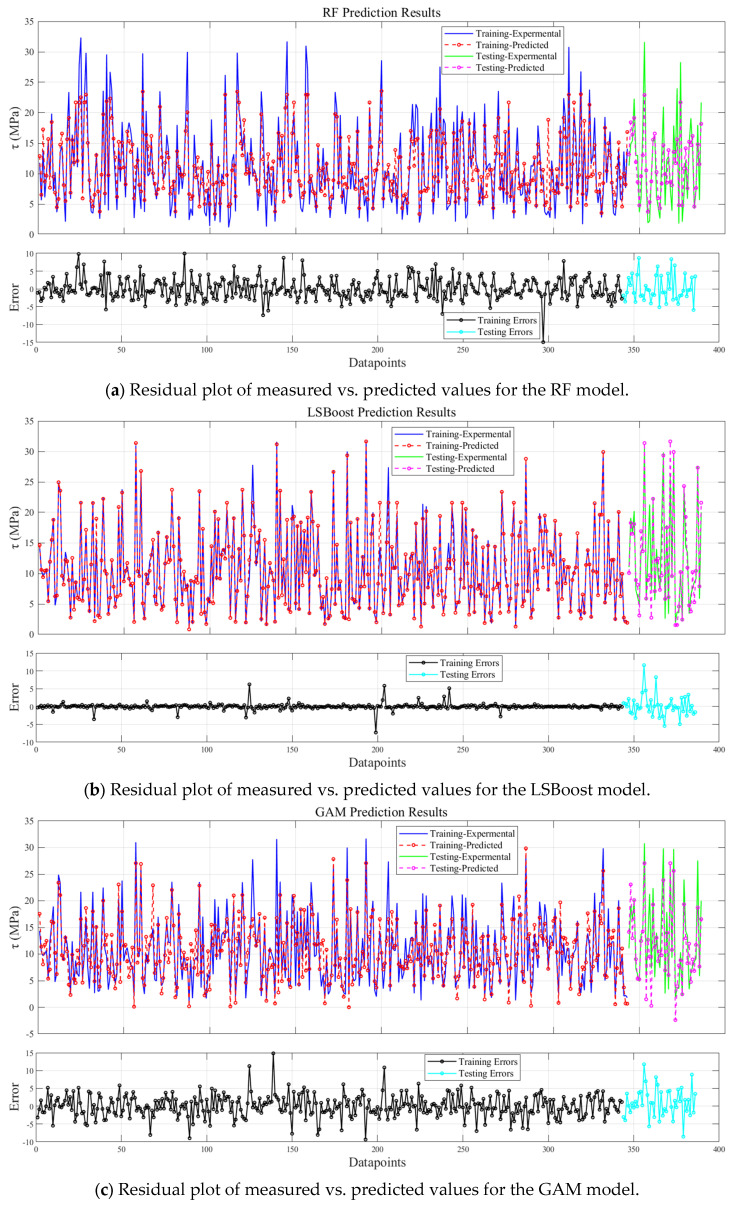

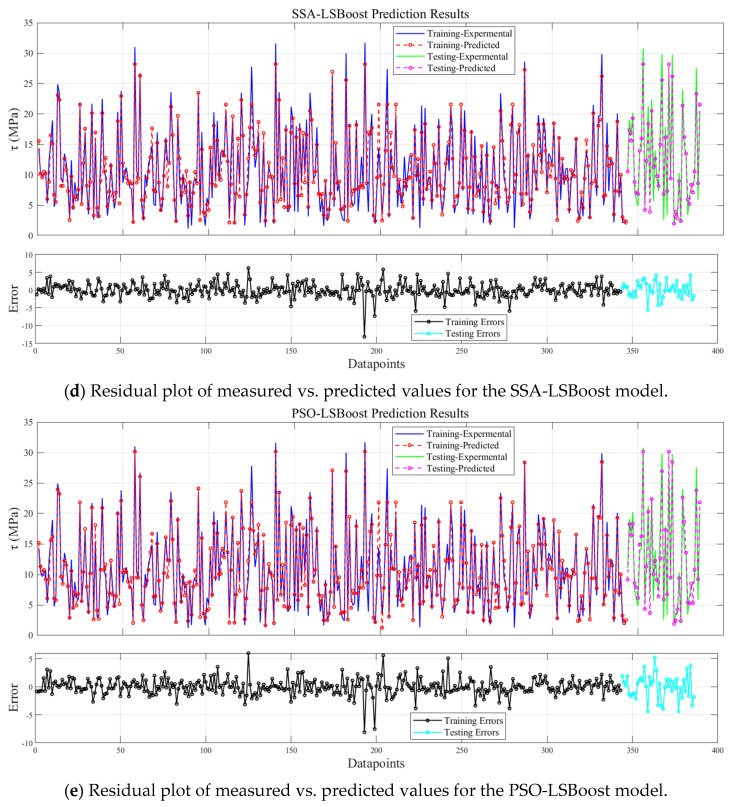
Residual plot of measured vs. predicted bond strength values for selected models.

Before training the models, all parameters were normalized, and the 429 data samples were randomly split into training, validation, and test sets in an 8:1:1 ratio. The training set consisted of 343 samples, while the validation and test sets each contained 43 samples. This study calculated the performance of 9 models, which included three traditional models—RF, LSBoost, and GAM—along with models optimized by two algorithms: SSA and PSO. Table 2 shows the error evaluation metrics for each model. Figure 7 illustrates the relationship between the predicted values and experimental values of *τ* for the 9 models, while Figure 8 shows the variations in *R*^2^ and MRE for the three models under different optimization algorithms. Figure 9 displays the residual plots for some of the models.

Figure 7a–c show that the prediction performance of the three traditional models—RF, LSBoost, and GAM—on *τ* was suboptimal. The *R^2^* for the RF model and GAM on the test set were only 0.756 and 0.749, respectively, with data points being relatively scattered from the y = x line and exhibiting large errors. Although the LSBoost model achieved a high *R*^2^ of 0.983 on the training set, its test set *R*^2^ was only 0.866, indicating overfitting to the training data. There are many reasons for overfitting, such as noisy data, high data variability, or the inclusion of feature factors that do not contribute to the model’s function [75].

Figure 7d–f demonstrate that after optimization with the SSA, the prediction performance of all three traditional models improved. The test set *R*^2^ for SSA-RF, SSA-LSBoost, and SSA-GAM increased to 0.826, 0.928, and 0.894, respectively, improving by 9.3%, 7.2%, and 19.4%. Among these, the SSA-LSBoost model had the best fit, with R^2^ values for both the training and test sets being close, indicating that the SSA optimization algorithm reduced the overfitting in the LSBoost model.

Figure 7g–i show the prediction performance of the three traditional models after optimization with the PSO algorithm. It can be observed that the test set *R*^2^ for PSO-RF and PSO-LSBoost increased further to 0.880 and 0.929, respectively, improving by 16.4% and 7.3%. This indicates that the PSO algorithm optimized the RF and LSBoost models better than the SSA. However, the PSO algorithm caused abnormal behavior in the GAM. Figure 8 visually reflects this phenomenon, as the *R*^2^ for the RF and LSBoost models consistently increased and *MRE* decreased after optimization with both SSA and PSO, while the *R*^2^ for the GAM decreased and the *MRE* increased after PSO. This proves that different optimization algorithms have a significant impact on model accuracy. This could be due to the mismatch between the optimization objective and the model characteristics, suggesting that the choice of algorithm should be considered based on the task properties, model architecture, and data distribution.

As shown in Figure 9, the residual range for the Random Forest (RF) model spans from −15 to 10, for LSBoost from −7.6 to 13.1, and for the Generalized Additive Model (GAM) from −9.9 to 15.1. Among these three conventional models, LSBoost exhibits the highest predictive accuracy. However, the residual range of LSBoost on the test set is substantially larger than that on the training set, indicating that, despite its high predictive performance, the model suffers from overfitting. After optimization with the SSA and PSO algorithms, the residual ranges on the training sets increased, while those on the test sets decreased. This demonstrates that both SSA and PSO effectively mitigate the overfitting issue inherent in the conventional models. Notably, PSO-LSBoost achieves the smallest residual range, from −8.1 to 5.9, indicating that it provides the highest prediction accuracy and robustness among all evaluated models.

### 4.2. Exhaustive Assessment of Forecasting Strategies

**Figure 10:**
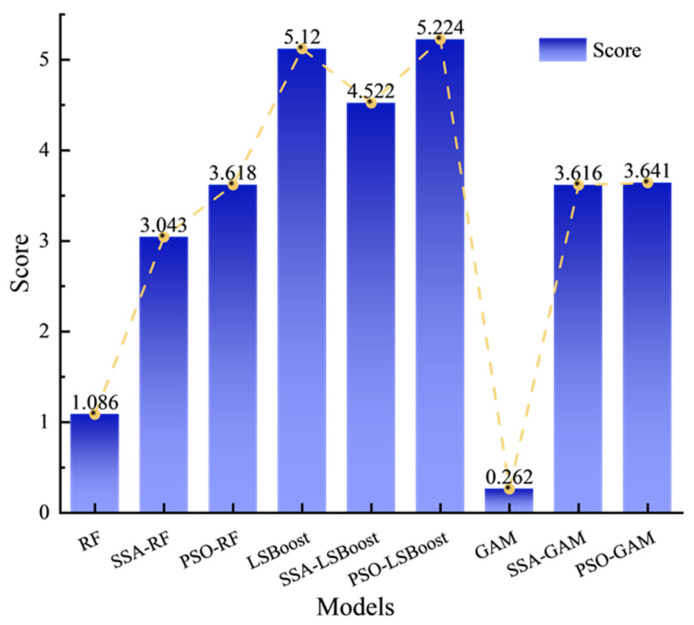
Total Score of Each Prediction Model.

To more rigorously compare the predictive performance of different models, this study adopts the model evaluation method proposed by Lv et al. [76], which quantifies prediction performance by standardizing multiple evaluation metrics during both the training and testing phases. A composite score is then computed to rank and compare models. This approach accounts for multiple criteria, thereby preventing any single metric from disproportionately influencing the evaluation results. Table 3 presents the normalized scores for all evaluation metrics using the Min–Max scaling method. Figure 10 shows the total scores of nine models for both the training and testing datasets. From Table 3, it is evident that LSBoost performs exceptionally well during the training phase, achieving the highest score on all three evaluation metrics, whereas GAM receives the lowest scores across the same metrics. In the testing phase, LSBoost’s performance declines significantly, obtaining only 2.12 points, while PSO-LSBoost achieves the highest score of 2.964 points. Figure 10 further illustrates that PSO-LSBoost attains the highest total scores in both training and testing phases, indicating that LSBoost optimized via PSO achieves the best overall performance. It is noteworthy that, although the total score of SSA-LSBoost is slightly lower than that of LSBoost, this discrepancy arises from the overfitting of LSBoost, which artificially inflates the training set scores. Consequently, in practical predictions, SSA-LSBoost may offer more balanced and reliable performance. Additionally, both RF and GAM demonstrate substantial improvements in total scores following optimization with the SSA and PSO algorithms.

To further highlight the contributions of this study, we compared our predictive model with other recent models developed for estimating the bond strength of reinforcement–concrete interfaces. Table 4 summarizes these recent studies. By comparing the coefficient of determination (*R*^2^) across different models, it is evident that the predictive performance of our model reaches a relatively high level among existing machine learning approaches. For example, the neural network model developed by X. Wang et al. [35] suffered from severe overfitting; although PSO improved its performance, the *R*^2^ on the training set remains slightly lower than that achieved in the present study. Similarly, the four models optimized via grid search by M. Ebrahimzadeh et al. [31] exhibited varying degrees of overfitting. The XGBoost model developed by M. Rahmati et al. [77] demonstrated high predictive accuracy, comparable to the performance of our model. Overall, this study not only achieves state-of-the-art predictive accuracy for reinforced concrete bond strength but also enriches the application of machine learning methods in this domain, providing a valuable reference for future research.

### 4.3. Comparison with Empirical Formulas

**Figure 11:**
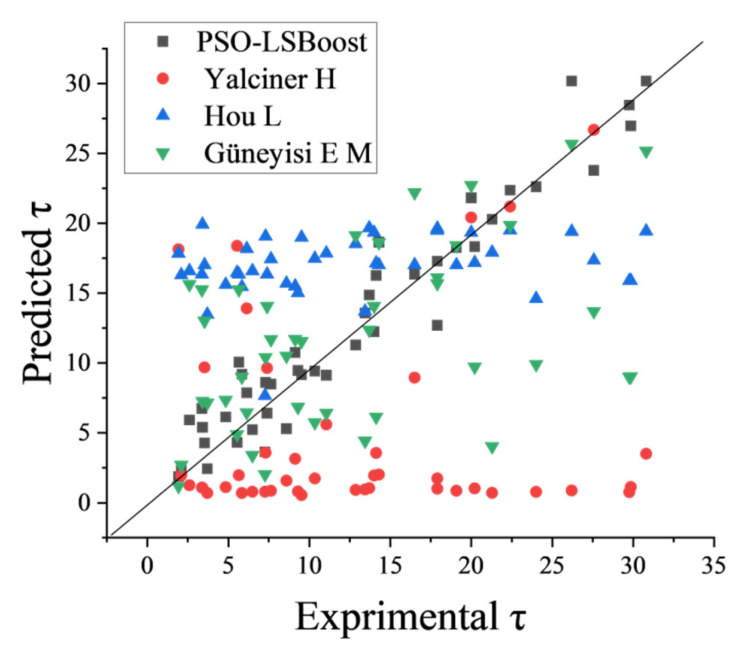
Distribution of prediction results for each empirical equation and PSO-LSBoost model.

Yalciner H et al. [41] established an empirical equation for the final bond strength based on the data from corrosion anchor bolt pullout tests using multiple linear regression, which depends on four factors: *D*_s_, *f*_c_, *C*_min_, and *C*_w_. The expressions are shown in Table 5. Hou L et al. [44], based on indoor accelerated corrosion and pullout test data, found that bond strength is greatly influenced by the corrosion rate. The bond strength follows a quadratic function with respect to the corrosion rate and exhibits a near-linear relationship with the ratio of rebar diameter to anchorage length. Therefore, considering these factors, a parabolic equation was proposed. After regression analysis, the undetermined coefficients were obtained, and the final empirical formula for anchor bolt bond strength is shown in Table 5. However, both models have the disadvantage of considering fewer factors and having limited experimental data. Güneyisi E M et al. [54] used Gene Expression Programming (GEP) to predict the corrosion anchor bolt bond strength. This model considers five parameters (*D*_s_, *L*_b_, *f*_c_, *ST*, *C*_w_) as independent variables and derives the expression for predicting *τ*, as shown in Table 5. The test set data from this study were input into the empirical equations mentioned in the three articles to predict the bond strength. Figure 11 shows a comparison of the prediction results of bond strength (*τ*) using the three empirical equations and the PSO-LSBoost model.

Among them, *D*_s_ is the rebar diameter, *L*_b_ is the anchorage length, *f*_c_ is the concrete strength, *ST* is the type of rebar, and *C*_w_ is the corrosion rate.

It can be observed that the empirical equation proposed by Yalciner H et al. generally underpredicts *τ* compared to the experimental values, with predictions concentrated in the range of 0 to 5 MPa. The empirical equation proposed by Hou L et al. also produces relatively concentrated predictions, mainly distributed near the average value of all data points, in the range of 15 to 20 MPa. This is because the empirical equations in both studies were fitted based on limited experimental data, with no more than 80 data points, and the experimental variables considered in both studies were few. For example, Hou L considered only three variables, while Yalciner H considered four variables, but the value of *f*_c_ was only 23 MPa and 51 MPa in two cases. Therefore, both models have certain limitations, and their predictions of *τ* are only applicable under the specific conditions in the literature, so the predicted results are naturally concentrated within a certain range. Güneyisi E M et al. used a machine learning model (GEP) to train a large amount of data and generate explicit mathematical expressions, thus avoiding the specificity issues inherent in traditional experimental methods. From Figure 8, it can be seen that the predicted values are roughly distributed along a sloped line, with higher accuracy than the first two empirical equations, but the data points are more scattered and the errors are larger. This is because the different choices of data samples, features, models, and optimization algorithms all have a significant impact on the model’s accuracy. The model collected only 218 data samples and 5 feature variables. Overall, the comparison analysis further confirms that the PSO-LSBoost model provides better predictions for *τ*.

## 5. SHAP Dependency Analysis

**Figure 12:**
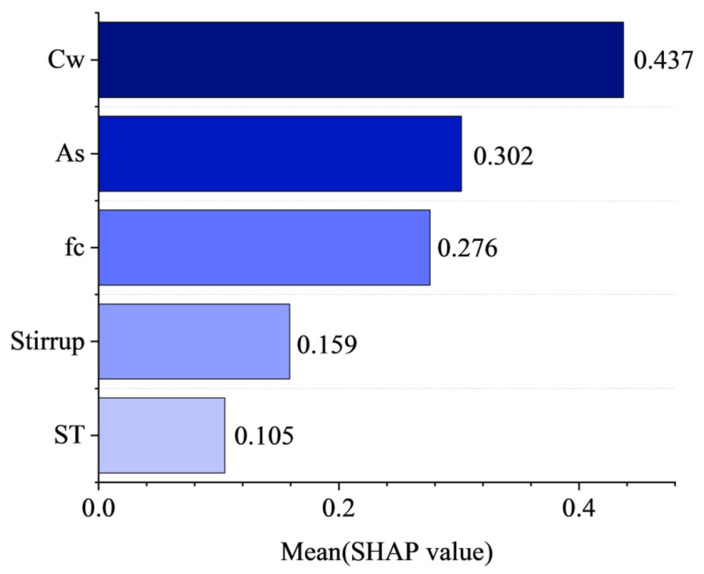
Mean absolute SHAP values of PSO-LSBoost model.

The bond strength prediction model constructed by machine learning is influenced by the complex interactions of multiple features. SHAP analysis provides consistent and interpretable quantitative metrics for the contribution of each feature variable to the model’s predictions [78,79]. This section performs SHAP analysis on the PSO-LSBoost model, which showed the best prediction performance. Figure 12 displays the average absolute SHAP values of the five feature variables for the PSO-LSBoost model’s predicted values of *τ*. The larger the SHAP value, the higher the contribution of the feature to the predicted value. The corrosion rate (*C*_w_) is the most influential feature on *τ*, followed by bond area (*A*_s_), concrete compressive strength (*f*_c_), presence of stirrups (*Stirrup*), and the type of rebar (*ST*), which has the lowest contribution. This is generally consistent with the conclusions from the earlier Spearman correlation analysis, indicating that *C*_w_, *A*_s_, and *f*_c_ are the most important features in predicting anchor bolt bond strength.

**Figure 13:**
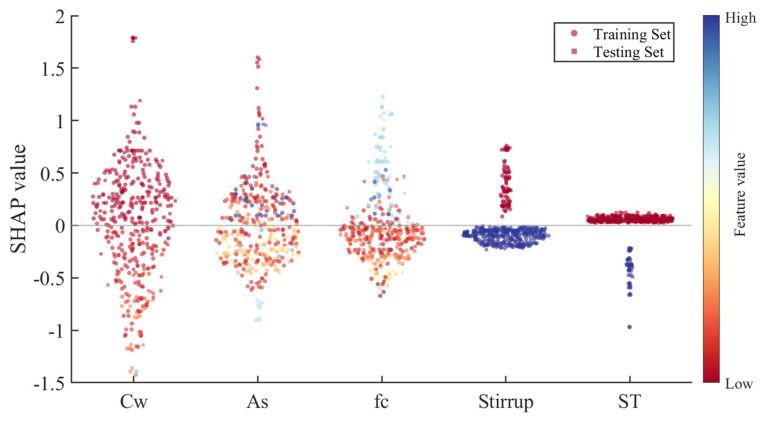
SHAP global explanation on PSO-LSBoost model.

Figure 13 shows the SHAP beeswarm plot for the PSO-LSBoost model, one of the most commonly used visual tools for SHAP analysis. This SHAP summary plot shows the impact of each feature on the model’s predictions across all samples. Each point represents a sample’s feature value and is colored on a spectrum from deep red (low values) to deep blue (high values). The horizontal position of a point corresponds to its SHAP value: points farther to the right increase the predicted *τ*, while those to the left decrease it. The *y*-axis denotes the SHAP value for each feature–sample pair. Points colored deep blue (high feature values) that appear toward the positive end of the *y*-axis indicate that larger values of that feature increase the predicted *τ*, signifying a positive correlation. Conversely, points colored deep red (low feature values) near the positive *y*-axis imply that smaller values of the feature also drive *τ* upward, reflecting a negative correlation. Moreover, a high density of blue points with positive SHAP values indicates a strong positive correlation, whereas a high density of red points with positive SHAP values indicates a strong negative correlation.

Taking the rebar type (*ST*) as an example, 1 represents threaded rebar and 2 represents smooth round rebar. The bond strength of smooth round rebar is generally lower than that of threaded rebar, meaning that as the value of *ST* increases, *τ* decreases, and *ST* is negatively correlated with *τ*. From Figure 10, it can be seen that the red scatter points (smaller values) are all distributed on the positive axis, while the blue scatter points (larger values) are all distributed on the negative axis, which aligns with theoretical expectations. Similarly, the feature “presence of stirrups” (*Stirrup*) follows the same pattern as *ST*. For the feature *f*_c_, the red points are mostly distributed on the negative axis, while the blue points are on the positive axis, indicating a positive correlation between fc and *τ*, which is also consistent with theory.

For the feature *A*_s_, both red and blue points are distributed on both the positive and negative axes, suggesting that *A*_s_ has a more complex relationship with *τ*, neither strictly positive nor negative. Some studies indicate that rebar diameter is negatively correlated with bond strength within a certain range, while anchorage length is positively correlated with bond strength within a specific range, but there exists a critical anchorage length beyond which the positive correlation no longer holds [80,81]. In this study, *A*_s_ combines the features of rebar diameter and anchorage length, which makes its relationship with *τ* more complex and not easily categorized as simply positive or negative. For the corrosion rate (*C*_w_), most deep red points are concentrated on the positive axis, while a certain number of red points are also distributed on the negative axis, suggesting that *C*_w_ is not solely negatively correlated with *τ*. In fact, some studies have shown that slight corrosion (before rust-induced cracking occurs) may temporarily increase bond strength due to the roughness of the rebar surface [82]. Overall, the SHAP analysis provides a good explanation of how each feature parameter contributes to the PSO-LSBoost model’s output value of *τ* and the variation in responses.

## 6. Conclusions

A total of 429 sets of pullout experimental data from anchor bolts or rebar–concrete specimens were collected, considering basic parameters such as rebar diameter, anchorage length, concrete compressive strength, and corrosion rate. The Sparrow Search Algorithm (SSA) and Particle Swarm Optimization (PSO) were used to optimize three traditional machine learning models, and the errors and reliability of each model were compared and analyzed. To evaluate performance, we compared the PSO-LSBoost model against three empirical equations and applied SHAP analysis to quantify each feature’s impact on the predictions, thereby clarifying how input variables drive model decisions. Based on these findings, we draw the following conclusions:

(1) Spearman correlation analysis showed that the corrosion rate (*C*_w_) had the highest correlation with the output value (*τ*). The rebar diameter (*D*_s_) and anchorage length (*L*_b_) were highly linearly correlated, while the cover thickness (*C*_min_) had a very low correlation with *τ* and was linearly correlated with several other features. After data preprocessing, five features were selected as the input parameters for the model.

(2) Combining RF, LSBoost, and GAM, three traditional machine learning models with SSA and PSO algorithms, a total of 9 models were computed. The three traditional models either had low prediction accuracy or exhibited overfitting. After optimizing the hyperparameters, the model accuracy generally improved. For this dataset, the PSO algorithm performed better than the SSA, except for the GAM, where the SSA outperformed the PSO algorithm. In conclusion, the PSO-LSBoost model provided the best prediction performance (Training set *R*^2^ = 0.95, Test set *R*^2^ = 0.93, Test set errors: *MAE* = 1.77, *MRE* = 0.22, *RMSE* = 2.21).

(3) Comparison of the PSO-LSBoost model with existing models and empirical formulas. The PSO-LSBoost model proposed in this study achieves predictive accuracy at a level higher than most existing models. Traditional experimental methods are constrained by time and funding, making it difficult to account for the combined effects of multiple complex variables, and the range of available data for each variable is often limited. Consequently, conventional empirical formulas generally provide accurate predictions only under specific conditions and lack broad applicability. In contrast, the PSO-LSBoost model developed in this study, based on a large dataset of 429 pull-out tests, demonstrates greater generalizability and higher predictive accuracy.

(4) SHAP analysis showed the following order of feature contributions to *τ*: *C*_w_ > *A*_s_ > *f*_c_ > *Stirrup* > *ST*. This suggests that during the design phase of anchor bolts, special attention should be given to corrosion protection measures for rebar, the area of the bond interface, and the strength grade of the anchoring material. The SHAP beeswarm plot effectively illustrated the response variation in each parameter to *τ*.

This study demonstrates that machine learning methods provide a feasible and effective approach for predicting *τ*. However, due to inconsistencies in the variables reported across the literature, only the seven most commonly observed parameters in anchor or reinforced concrete design were comprehensively compiled and analyzed. In practical engineering applications, anchor performance may also be influenced by additional factors, such as temperature, moisture content, freeze–thaw cycles, anchor material, and the mix proportions of concrete or mortar. Future research should expand the range of influencing variables to further enhance the predictive accuracy of the models. Although this study utilizes a relatively large dataset, it exhibits uneven data distributions, which may affect the final prediction results. Given the current limitations in available literature, addressing this issue will require additional time and data collection. This limitation also highlights a potential direction for future research.

## Figures and Tables

**Table 1:** Statistical description of variables.

Variables	Notation	Min	Max	Mean	Standard Deviation	Median
Diameter of rebar (mm)	*D*_s_	10	25	16.5	0.41	16
Bond length (mm)	*L_b_*	20	200	87.26	3.03	80
Compressive strength (MPa)	*f_c_*	20.7	64.9	34.28	0.30	30
Concrete cover (mm)	*C_min_*	15	147.5	61.62	1.25	65
Steel type	*ST*	–	–	–	–	–
Whether to provide stirrups	*Stirrup*	–	–	–	–	–
Corrosion level (%)	*C*_w_	0	80	3.96	0.11	2.09
Bond strength (MPa)	*τ*	1.18	32.35	11.04	0.48	9.5

**Table 2:** Error statistics.

Model	FR		SSA-RF		PSO-RF	
	Training	Testing	Training	Testing	Training	Testing
*R* ^2^	0.816	0.756	0.890	0.826	0.900	0.880
*MAE*	2.292	2.939	1.664	2.327	1.556	2.230
*MRE*	33.2%	39.3%	24.6%	30.0%	23.8%	28.1%
*RMSE*	2.969	3.617	2.303	3.055	2.093	2.875
Model	LSBoost		SSA-LSBoost		PSO-LSBoost	
	Training	Testing	Training	Testing	Training	Testing
*R* ^2^	0.983	0.866	0.905	0.928	0.949	0.929
*MAE*	0.379	2.052	1.482	1.796	1.058	1.766
*MRE*	4.4%	24.1%	22.6%	22.6%	15.4%	22.3%
*RMSE*	0.869	3.046	2.042	2.237	1.492	2.214
Model	GAM		SSA-GAM		PSO-GAM	
	Training	Testing	Training	Testing	Training	Testing
*R* ^2^	0.777	0.749	0.885	0.894	0.947	0.685
*MAE*	2.410	3.227	1.608	2.196	1.125	2.157
*MRE*	36.4%	41.3%	24.1%	28.0%	14.4%	42.2%
*RMSE*	3.124	4.168	2.249	2.709	1.575	2.675

**Table 3:** Evaluation of comprehensive forecasting results for each model.

Dataset	Models	Evaluation Metrics and Scores						
		MAE	Score	RMSE	Score	*R* ^2^	Score	Total Score
Train	RF	2.292	0.058	2.969	0.069	0.816	0.189	0.316
	SSA-RF	1.664	0.367	2.303	0.364	0.890	0.549	1.280
	PSO-RF	1.556	0.420	2.093	0.457	0.900	0.597	1.475
	LSBoost	0.379	1	0.869	1	0.983	1	3
	SSA-LSBoost	1.482	0.457	2.042	0.480	0.905	0.621	1.558
	PSO-LSBoost	1.058	0.666	1.492	0.724	0.949	0.835	2.224
	GAM	2.410	0	3.124	0	0.777	0	0
	SSA-GAM	1.608	0.395	2.249	0.388	0.885	0.524	1.307
	PSO-GAM	1.125	0.633	1.575	0.687	0.947	0.825	2.145
Test	RF	2.939	0.197	3.617	0.282	0.756	0.291	0.770
	SSA-RF	2.327	0.616	3.055	0.570	0.826	0.578	1.763
	PSO-RF	2.230	0.682	2.875	0.662	0.880	0.799	2.143
	LSBoost	2.052	0.804	3.046	0.574	0.866	0.742	2.120
	SSA-LSBoost	1.796	0.979	2.237	0.988	0.928	0.996	2.964
	PSO-LSBoost	1.766	1	2.214	1	0.929	1	3
	GAM	3.227	0	4.168	0	0.749	0.262	0.262
	SSA-GAM	2.196	0.706	2.709	0.747	0.894	0.857	2.309
	PSO-GAM	2.157	0.732	2.675	0.764	0.685	0	1.496

**Table 4:** Performance Comparison (*R*^2^) of Bond Strength Prediction Models for Concrete and Steel Reinforcement.

Study	Predictive Model	Target Variable	R^2^ (Train Set)	R^2^ (Test Set)
This study [35]	PSO-LSBoost	Bond Strength (*τ*)	0.95	0.93
	ANN		0.91	0.62
	GA-ANN		0.92	0.87
	PSO-ANN		0.93	0.93
[31]	Grid Search for DT		0.90	0.85
	Grid Search for RF		0.97	0.86
	Grid Search for GB		0.98	0.89
	Grid Search for XGB		0.99	0.90
[77]	M5P		0.94	0.86
	RF		0.89	0.90
	XGBoost		0.95	0.93

**Table 5:** Empirical formulas for predicting bond strength of corroded anchor bolts.

Reference	Factors Influencing	Empirical Formula
Yalciner H et al. [41]	*D*_s_, *f*_c_, *C*_min_, *C*_w_	$\tau_{u}=0.40551f_{c}-0.25306\left(\frac{C_{min}}{D_{s}}\right)+0.97926C_{w}\;\frac{C_{min}}{D_{s}}<2,$ $\tau_{u}=e^{0.01572f_{c}-0.22957\left(\frac{C_{min}}{D_{s}}\right)+0.13946C_{w}+1.75913},\frac{C_{min}}{D_{s}}\geq 2$
Hou L et al. [44]	*D*_s_, *L*_b_, *C*_w_	$\tau_{u}=\alpha [A{C_{w}}^{2}+BC_{w}+C]({\frac{D_{s}}{L_{b}})}^{\beta }$ $\tau_{u}=0.335[-0.124{C_{w}}^{2}+1.183C_{w}+93.504]({\frac{D_{s}}{L_{b}})}^{0.379}$
Güneyisi E M et al. [54]	*D*_s_, *L*_b_, *f*_c_, *ST*, *C*_w_	$\tau_{u}=\tau_{1}+\tau_{2}+\tau_{3}+\tau_{4}+\tau_{5}+\tau_{6}$ $\tau_{1}=tan[cos(\frac{2C_{w}}{\sqrt{D_{s}}}+f_{c})]$ $\tau_{2}=tan[cos({\left(cosf_{c}\right)}^{5}+C_{w}+L_{b}-0.603425)]$ $\tau_{3}=tan[tanh(f_{c}\times C_{w}+\sqrt[{3}]{C_{min}}-D_{s})]$ $\tau_{4}=tan[cos(C_{min}-\sqrt{ST}\times D_{s}+C_{w})]$ $\tau_{5}=\frac{33.59253-C_{w}+f_{c}}{C_{w}+\sqrt[{3}]{L_{b}}}$ $\tau_{6}=tan[cos(\sqrt[{3}]{C_{w}}-\tan\left(C_{min}\times ST-L_{b}\right))]$

## Data Availability

Data will be made available on request.

## Appendix A. The Collected Dataset

**Table A1:** The Collected Dataset.

Reference	No.	*D*_s_ (mm)	*L*_b_ (mm)	*f*_c_ (MPa)	*C*_min_ (mm)	*ST*	*Stirrup*	*C*_w_ (%)	*Τ* (MPa)
[40]	1	16	80	42.25	67	2	2	0	10.08
	2	16	80	42.25	67	2	2	5.81	5.91
	3	16	80	42.25	67	2	2	7.77	6.44
	4	16	80	42.25	67	2	2	8.84	7.54
	5	16	80	42.25	67	2	2	11.25	6.49
	6	16	80	42.25	67	2	2	12.63	5.63
	7	16	80	42.25	67	2	2	13.68	6.67
	8	16	80	42.25	67	2	2	14.67	3.47
	9	16	80	42.25	67	2	2	16.13	4.04
	10	16	80	42.25	67	2	2	17.33	10.61
	11	16	80	42.25	67	2	2	20.88	2.71
	12	16	80	42.25	67	2	2	25.68	7.27
[41]	13	14	50	23	15	1	2	0	9.1
	14	14	50	23	15	1	2	0	9.4
	15	14	50	23	15	1	2	0	9.2
	16	14	50	23	30	1	2	0	14
	17	14	50	23	30	1	2	0	12.3
	18	14	50	23	30	1	2	0	13.5
	19	14	50	23	45	1	2	0	12.1
	20	14	50	23	45	1	2	0	17.3
	21	14	50	23	45	1	2	0	15
	22	14	50	23	15	1	2	8.9	3.7
	23	14	50	23	15	1	2	4.1	13
	24	14	50	23	15	1	2	2.47	11.2
	25	14	50	23	15	1	2	2.72	11.7
	26	14	50	23	15	1	2	4.32	12.2
	27	14	50	23	15	1	2	4.33	12.2
	28	14	50	23	15	1	2	4.09	13
	29	14	50	23	15	1	2	6.51	3.2
	30	14	50	23	15	1	2	14.52	2.1
	31	14	50	23	30	1	2	1.37	18
	32	14	50	23	30	1	2	3.45	9.6
	33	14	50	23	30	1	2	5.56	3.3
	34	14	50	23	30	1	2	1.4	17.9
	35	14	50	23	30	1	2	1.69	16.9
	36	14	50	23	30	1	2	1.6	17
	37	14	50	23	30	1	2	3.57	8.9
	38	14	50	23	30	1	2	5.36	3.7
	39	14	50	23	30	1	2	16.65	2.1
	40	14	50	23	45	1	2	0.69	19.1
	41	14	50	23	45	1	2	1.69	13.4
	42	14	50	23	45	1	2	2.66	12.4
	43	14	50	23	45	1	2	0.68	17.9
	44	14	50	23	45	1	2	0.66	18.9
	45	14	50	23	45	1	2	0.84	18.3
	46	14	50	23	45	1	2	0.88	18.2
	47	14	50	23	45	1	2	1.6	13.7
	48	14	50	23	45	1	2	3.81	1.3
	49	14	50	23	15	1	2	18.75	4.3
	50	14	50	23	15	1	2	8.9	3
	51	14	50	23	15	1	2	14.66	2
	52	14	50	23	30	1	2	6.87	6.5
	53	14	50	23	30	1	2	17.33	1.8
	54	14	50	23	30	1	2	6.4	5.5
	55	14	50	23	45	1	2	6.27	3.2
	56	14	50	23	45	1	2	0.68	18
	57	14	50	23	45	1	2	3.81	1.3
	58	14	50	51	15	1	2	0	19.6
	59	14	50	51	15	1	2	0	14.3
	60	14	50	51	15	1	2	0	20
	61	14	50	51	30	1	2	0	20.9
	62	14	50	51	30	1	2	0	21.7
	63	14	50	51	30	1	2	0	21
	64	14	50	51	45	1	2	0	21.2
	65	14	50	51	45	1	2	0	27.4
	66	14	50	51	45	1	2	0	27.8
	67	14	50	51	15	1	2	1.33	18.5
	68	14	50	51	15	1	2	7.48	3.5
	69	14	50	51	15	1	2	4.47	6.3
	70	14	50	51	15	1	2	0.77	22.3
	71	14	50	51	15	1	2	0.8	22.4
	72	14	50	51	15	1	2	0.9	21.7
	73	14	50	51	15	1	2	0.94	21.5
	74	14	50	51	15	1	2	7.56	3.5
	75	14	50	51	15	1	2	3.3	7.5
	76	14	50	51	30	1	2	0	20.4
	77	14	50	51	30	1	2	5.14	6.2
	78	14	50	51	30	1	2	5.46	2.4
	79	14	50	51	30	1	2	0.65	23.8
	80	14	50	51	30	1	2	0.68	3.9
	81	14	50	51	30	1	2	0.77	23.5
	82	14	50	51	30	1	2	0.77	23.4
	83	14	50	51	30	1	2	1.7	14
	84	14	50	51	30	1	2	4.45	4.2
	85	14	50	51	45	1	2	0	28.3
	86	14	50	51	45	1	2	2.69	7.6
	87	14	50	51	45	1	2	0.34	26.2
	88	14	50	51	45	1	2	0.31	31.6
	89	14	50	51	45	1	2	0.4	31
	90	14	50	51	45	1	2	0.41	30.8
	91	14	50	51	45	1	2	4.73	3
	92	14	50	51	45	1	2	4.38	3.4
	93	14	50	51	45	1	2	4.17	3.9
	94	14	50	51	15	1	2	8.95	3
	95	14	50	51	15	1	2	6.9	8
	96	14	50	51	15	1	2	3.41	6.8
	97	14	50	51	30	1	2	9.9	5.9
	98	14	50	51	30	1	2	4.86	1.7
	99	14	50	51	30	1	2	1.72	13.8
	100	14	50	51	45	1	2	0.34	26.9
	101	14	50	51	45	1	2	0.34	31.7
	102	14	50	51	45	1	2	3.08	6.1
[42]	103	13	37.1	28.3	68.5	1	2	0	14.7
	104	13	36.6	28.3	68.5	1	2	0	17
	105	13	37.9	28.3	68.5	1	2	0	14
	106	13	37.2	28.3	68.5	1	2	0.1	20.1
	107	13	37.1	28.3	68.5	1	2	0.5	17.4
	108	13	37.4	28.3	68.5	1	2	1	20
	109	13	37	28.3	68.5	1	2	1.1	14.5
	110	13	36.7	28.3	68.5	1	2	1.2	16.2
	111	13	37.1	28.3	68.5	1	2	1.4	17.9
	112	13	38	28.3	68.5	1	2	0.9	16.4
	113	13	39.1	28.3	68.5	1	2	2.5	20.6
	114	13	37	28.3	68.5	1	2	0.2	15.9
	115	13	36.7	28.3	68.5	1	2	0.8	18.5
	116	13	37.1	28.3	68.5	1	2	1.9	20.3
	117	13	37.1	28.3	68.5	1	2	0.9	14.1
	118	13	36.5	28.3	68.5	1	2	0.5	16.7
	119	13	37	28.3	68.5	1	2	1.4	19
	120	13	37.8	28.3	68.5	1	2	2.2	14.4
	121	13	36.1	28.3	68.5	1	2	0.6	16.1
	122	13	35.9	28.3	68.5	1	2	0.7	15.4
	123	13	36.8	28.3	68.5	1	2	1.9	15.9
[43]	124	18	100	41.9	66	1	2	0	5.2
	125	18	100	41.9	66	1	2	0.65	4.78
	126	18	100	41.9	66	1	2	1.21	9.41
	127	18	100	41.9	66	1	2	1.89	5.33
	128	18	100	41.9	66	1	2	3.93	4.26
	129	18	100	41.9	66	1	2	5.95	2.19
	130	18	100	38.4	66	1	2	0	3.37
	131	18	100	38.4	66	1	2	0.9	6.39
	132	18	100	38.4	66	1	2	1.32	2.6
	133	18	100	38.4	66	1	2	1.68	4.33
	134	18	100	38.4	66	1	2	4.02	2.11
	135	18	100	38.4	66	1	2	6.18	1.18
	136	18	100	41.9	66	1	1	0	7.34
	137	18	100	41.9	66	1	1	1.83	8.98
	138	18	100	41.9	66	1	1	2.66	11.05
	139	18	100	41.9	66	1	1	3.69	9.6
	140	18	100	38.4	66	1	1	0	5.65
	141	18	100	38.4	66	1	1	1.96	9.94
	142	18	100	38.4	66	1	1	2.89	7.27
	143	18	100	38.4	66	1	1	3.99	7.48
[44]	144	16	48	56.6	67	1	2	0	19.84
	145	16	80	56.6	67	1	2	0	14.93
	146	16	112	56.6	67	1	2	0	10.67
	147	16	48	45.5	67	1	2	0	20.37
	148	16	80	45.5	67	1	2	0	19.08
	149	16	112	45.5	67	1	2	0	13.87
	150	16	48	45.5	67	1	2	4.81	19.68
	151	16	80	45.5	67	1	2	4.41	18.38
	152	16	112	45.5	67	1	2	4.43	14.53
	153	16	48	45.5	67	1	2	9.25	21.43
	154	16	80	45.5	67	1	2	9.67	18.95
	155	16	112	45.5	67	1	2	7.32	14.57
	156	16	48	45.5	67	1	2	14.91	18.23
	157	16	80	45.5	67	1	2	13.54	15.51
	158	16	112	45.5	67	1	2	14.12	13.44
[45]	159	12	102	30	69	1	1	0	15.86
	160	12	102	30	69	1	1	2	16.25
	161	12	102	30	69	1	1	2.5	16.38
	162	12	102	30	69	1	1	2.6	17.81
	163	12	102	30	69	1	1	4	18.59
	164	12	102	30	69	1	1	5	17.03
	165	12	102	30	69	1	1	5.5	16.38
	166	12	102	30	69	1	1	6.5	13.52
	167	12	102	30	69	1	1	7	10.79
	168	12	102	30	69	1	1	8	4.94
	169	12	102	30	69	1	1	8.5	4.68
	170	12	102	30	69	1	1	11.5	3.38
	171	12	102	30	69	1	1	15.5	3.12
	172	12	102	30	69	1	1	20.5	2.73
	173	12	102	30	69	1	1	32.5	2.68
	174	12	102	30	69	1	1	48	2.63
	175	12	102	30	69	1	1	60.2	2.5
	176	12	102	30	69	1	1	80	2.44
[46]	177	10	20	36.88	70	2	2	0	1.69
	178	25	50	36.88	62.5	2	2	0	2.11
	179	10	20	36.88	70	1	2	0	9.02
	180	25	50	36.88	62.5	1	2	0	12.26
[47]	181	10	51.6	51.15	45	1	2	0	26.78
	182	10	48.1	53.87	45	1	2	0	32.35
	183	10	49.2	51.78	45	1	2	0	28.61
	184	10	56.3	51.15	45	1	2	2.7	23.58
	185	10	46.2	53.87	45	1	2	2.8	29.57
	186	10	48.3	51.78	45	1	2	2.1	26.71
	187	10	52.7	51.15	45	1	2	5.6	20.21
	188	10	51.3	53.87	45	1	2	5.5	27.57
	189	10	49.3	51.78	45	1	2	7.6	23.52
	190	10	51.8	51.15	45	1	2	12.2	16.99
	191	10	49.8	53.87	45	1	2	11.5	24.9
	192	10	43.3	51.78	45	1	2	9.8	21.29
[48]	193	20	100	61.8	65	1	2	0	16.5
	194	20	100	61.8	65	1	2	0.57	19.63
	195	20	100	61.8	65	1	2	1.96	12.96
	196	20	100	61.8	65	1	2	2.9	5.31
	197	20	100	64.9	65	1	2	0	16.4
	198	20	100	64.9	65	1	2	0.75	13.64
	199	20	100	64.9	65	1	2	1.71	11.04
	200	20	100	64.9	65	1	2	2.54	6.57
	201	20	100	52.6	65	1	2	0	14.3
	202	20	100	52.6	65	1	2	0.67	12.33
	203	20	100	52.6	65	1	2	1.37	9.87
	204	20	100	52.6	65	1	2	2.7	7.63
	205	20	100	39.7	65	1	2	0	13.01
	206	20	100	39.7	65	1	2	0.44	7.98
	207	20	100	39.7	65	1	2	1.42	6.19
	208	20	100	39.7	65	1	2	2.63	4.8
	209	20	100	40.6	65	1	2	0	12.31
	210	20	100	40.6	65	1	2	1.7	5.31
	211	20	100	40.6	65	1	2	2.54	4.79
	212	20	100	32	65	1	2	0	11.61
	213	20	100	32	65	1	2	0.73	5.75
	214	20	100	32	65	1	2	2.1	5.02
	215	20	100	32	65	1	2	2.9	4.49
[49]	216	20	200	35.5	20	1	1	0	9.6
	217	20	200	35.5	20	1	1	1.05	11.3
	218	20	200	35.5	20	1	1	2.95	10.25
	219	20	200	35.5	20	1	1	5.8	9.14
	220	20	200	35.5	20	1	1	8.95	8.79
[50]	221	20	80	52.1	60	1	2	0	15.4
	222	20	80	52.1	60	1	2	0	21.2
	223	20	80	52.1	60	1	2	0	21.4
	224	20	80	52.1	60	1	2	0.1	15.8
	225	20	80	52.1	60	1	2	2	12
	226	20	80	52.1	60	1	2	2.2	13
	227	20	80	52.1	60	1	2	3.5	11.4
	228	20	80	52.1	60	1	2	4.4	12
	229	20	80	52.1	60	1	2	5.8	6.2
	230	20	80	52.1	60	1	2	6.8	8.5
	231	20	80	52.1	60	1	2	9	7
	232	20	80	52.1	60	1	2	9	7.5
	233	20	80	52.1	60	2	2	0	4
	234	20	80	52.1	60	2	2	0	6.2
	235	20	80	52.1	60	2	2	0.7	10.7
	236	20	80	52.1	60	2	2	1.2	14.2
	237	20	80	52.1	60	2	2	3.25	7.6
	238	20	80	52.1	60	2	2	3.5	10.7
	239	20	80	52.1	60	2	2	4.1	7.3
	240	20	80	52.1	60	2	2	6.8	8
[51]	241	19	177.8	28	79.4	1	2	0	6.32
	242	19	178.8	28	79.4	1	2	0	5.79
	243	19	179.8	28	79.4	1	2	0.72	7.67
	244	19	180.8	28	79.4	1	2	0.72	7.13
	245	19	181.8	28	79.4	1	2	0.98	8.41
	246	19	182.8	28	79.4	1	2	1.23	4.91
	247	19	183.8	28	79.4	1	2	1.44	3.1
	248	19	184.8	28	79.4	1	2	1.7	3.79
	249	19	185.8	28	79.4	1	2	2.21	3.7
	250	19	186.8	28	79.4	1	2	2.88	2.09
	251	19	187.6	28	79.4	1	2	5.19	1.41
[52]	252	12	80	22.13	44	2	2	0.27	2.65
	253	12	80	22.13	44	2	2	0.29	3.23
	254	12	80	22.13	44	2	2	0.92	5.79
	255	12	80	22.13	44	2	2	1.13	5.84
	256	12	80	22.13	44	2	2	0.78	7.41
	257	12	80	22.13	44	2	2	1.47	8.63
	258	12	80	22.13	44	2	2	1.85	7.3
	259	12	80	22.13	44	2	2	1.5	7.96
	260	12	80	22.13	44	2	2	1.99	9.29
	261	12	80	22.13	44	2	2	1.04	10.26
	262	12	80	22.13	44	2	2	2.75	5.97
	263	12	80	22.13	44	2	2	2.43	4.84
	264	12	80	22.13	44	2	2	4.77	3.75
	265	12	80	22.13	44	2	2	5.01	1.63
	266	12	80	22.13	44	1	2	0.12	8.92
	267	12	80	22.13	44	1	2	0.16	9.49
	268	12	80	22.13	44	1	2	0.24	7.37
	269	12	80	22.13	44	1	2	0.32	8.5
	270	12	80	22.13	44	1	2	0.43	8.39
	271	12	80	22.13	44	1	2	0.62	10.62
	272	12	80	22.13	44	1	2	0.81	11.35
	273	12	80	22.13	44	1	2	1.4	9.99
	274	12	80	22.13	44	1	2	2.54	9.95
	275	12	80	22.13	44	1	2	3.75	8.59
	276	12	80	22.13	44	1	2	4.45	8.7
	277	12	80	22.13	44	1	2	5.68	5.97
	278	12	80	22.13	44	1	2	7.5	4.64
	279	12	80	22.13	44	1	2	9.72	1.66
[53]	280	14	60	21	30	1	1	0	6.63
	281	14	60	21	30	1	1	1.9	8.54
	282	14	60	21	30	1	1	3.6	10.74
	283	14	60	21	30	1	2	0	4.31
	284	14	60	21	30	1	2	3.1	5.87
	285	14	60	21	30	1	2	3.5	4.98
	286	14	60	21	20	1	1	0	4.02
	287	14	60	21	20	1	1	3.3	3.98
	288	14	60	21	40	1	1	0	4.76
	289	14	60	21	40	1	1	4.1	10.14
[54]	290	25	160	30	147.5	1	2	0	9.84
	291	25	160	30	147.5	1	2	0	10.48
	292	25	160	30	147.5	1	2	0	11.91
	293	25	160	30	147.5	1	2	5.62	7.68
	294	25	160	30	147.5	1	2	5.84	8.09
	295	25	160	30	147.5	1	2	3.4	9.68
	296	25	160	30	147.5	1	2	3.05	7.67
	297	25	160	30	147.5	1	2	5.58	5.17
	298	25	160	30	147.5	1	2	5.19	7.17
	299	25	160	30	147.5	1	2	4.15	6.1
	300	25	160	30	147.5	1	2	5.19	9.03
	301	25	160	30	147.5	1	2	6.82	6.48
	302	25	160	30	147.5	1	2	0	9.13
	303	25	160	30	147.5	1	2	0	10
	304	25	160	30	147.5	1	2	3.2	11.2
	305	25	160	30	147.5	1	2	4.69	7.89
	306	25	160	30	147.5	1	2	4.35	8.08
	307	25	160	30	147.5	1	2	3.88	11.32
	308	25	160	30	147.5	1	2	4.45	9.04
	309	25	160	30	147.5	1	2	4.39	7.64
	310	25	160	30	147.5	1	2	4.52	7.28
	311	25	160	30	147.5	1	2	0	10.43
	312	25	160	30	147.5	1	2	0	10.92
	313	25	160	30	147.5	1	2	0	9.69
	314	25	160	30	147.5	1	2	2.09	8.7
	315	25	160	30	147.5	1	2	1.78	13.63
	316	25	160	30	147.5	1	2	3.09	4.93
	317	25	160	30	147.5	1	2	1.31	10.89
	318	25	160	30	147.5	1	2	2.22	5.91
	319	25	160	30	147.5	1	2	3.16	13.02
	320	25	160	30	147.5	1	2	0.79	9.36
	321	25	160	30	147.5	1	2	4.1	7.93
	322	25	160	30	147.5	1	2	5.33	3.89
	323	25	160	30	147.5	1	2	4.13	8.7
[55]	324	18	100	24.81	30	2	2	0	3.49
	325	18	100	24.81	30	2	2	1.62	6.39
	326	18	100	24.81	30	2	2	1.78	4.61
	327	18	100	24.81	30	2	2	3.38	3.68
	328	18	100	24.81	30	2	2	7.86	2.12
	329	20	100	24.81	30	2	2	0	3.53
	330	20	100	24.81	30	2	2	2.52	5.48
	331	20	100	24.81	30	2	2	4.13	3.89
	332	20	100	24.81	30	2	2	7.51	2.79
	333	20	100	24.81	30	2	2	7.65	2.02
	334	20	100	24.81	30	2	2	10.41	1.83
	335	22	100	24.81	30	2	2	0	3.71
	336	22	100	24.81	30	2	2	3.34	3.67
	337	22	100	24.81	30	2	2	4.2	2.75
	338	22	100	24.81	30	2	2	6.55	2.24
	339	22	100	24.81	30	2	2	6.55	1.96
	340	22	100	24.81	30	2	2	8.6	1.93
	341	18	100	24.81	30	1	2	0	7.39
	342	18	100	24.81	30	1	2	3.27	8.67
	343	18	100	24.81	30	1	2	3.76	7.96
	344	18	100	24.81	30	1	2	4.95	6.67
	345	18	100	24.81	30	1	2	8.93	5.54
	346	18	100	24.81	30	1	2	9.06	5.16
	347	20	100	24.81	30	1	2	0	7.73
	348	20	100	24.81	30	1	2	2.48	8.18
	349	20	100	24.81	30	1	2	3.84	9.05
	350	20	100	24.81	30	1	2	4.32	5.14
	351	20	100	24.81	30	1	2	6.53	4.24
	352	20	100	24.81	30	1	2	8.29	3.66
	353	22	100	24.81	30	1	2	0	6.67
	354	22	100	24.81	30	1	2	1.43	7.54
	355	22	100	24.81	30	1	2	2.93	7.72
	356	22	100	24.81	30	1	2	4.28	6.14
	357	22	100	24.81	30	1	2	8.75	3.98
[56]	358	10	40	30	70	1	1	0	12.86
	359	14	60	30	68	1	1	0	11.02
	360	10	40	25	70	1	1	0	12.22
	361	14	60	25	68	1	1	0	10.47
	362	10	40	30	70	1	1	2.15	10.03
	363	10	40	30	70	1	1	2.77	9.52
	364	10	40	30	70	1	1	3.38	8.88
	365	14	60	30	68	1	1	1.93	8.92
	366	14	60	30	68	1	1	2.04	8.38
	367	14	60	30	68	1	1	2.64	7.94
	368	10	40	25	70	1	1	2.21	12
	369	10	40	25	70	1	1	3.32	11.78
	370	10	40	25	70	1	1	4.35	8.18
	371	14	60	25	68	1	1	1.02	10.32
	372	14	60	25	68	1	1	1.98	10.169
	373	14	60	25	68	1	1	2.62	7.42
[57]	374	16	150	28	92	1	2	0	6
	375	16	150	28	92	1	2	20.88	2.65
	376	16	150	28	92	1	2	20.42	2.6
	377	16	150	28	92	1	2	19.98	2.7
	378	20	150	28	90	1	2	0	13
	379	20	150	28	90	1	2	3.63	9.5
	380	20	150	28	90	1	2	3.63	9.3
	381	20	150	28	90	1	2	3.89	9.11
	382	25	150	28	87.5	1	2	0	17
	383	25	150	28	87.5	1	2	1.42	14.2
	384	25	150	28	87.5	1	2	1.26	14.3
	385	25	150	28	87.5	1	2	1.75	14.35
	386	16	150	28	92	1	1	0	21
	387	16	150	28	92	1	1	0.87	19
	388	16	150	28	92	1	1	1.27	19.2
	389	16	150	28	92	1	1	0.47	19.3
	390	20	150	28	90	1	1	0	24
	391	20	150	28	90	1	1	0.12	23.5
	392	20	150	28	90	1	1	0.15	23.6
	393	20	150	28	90	1	1	0.17	23.4
	394	25	150	28	87.5	1	1	0	30
	395	25	150	28	87.5	1	1	0.005	29.87
	396	25	150	28	87.5	1	1	0.003	29.75
	397	25	150	28	87.5	1	1	0.002	29.85
[58]	398	12	70	29	69	1	2	0	11.21
	399	12	70	29	69	1	2	0.1	14.13
	400	12	70	29	69	1	2	0.46	17.56
	401	12	70	29	69	1	2	0.8	16.74
	402	12	70	29	69	1	2	0.88	15.42
	403	12	70	29	69	1	2	0.94	11.74
	404	12	70	29	69	1	2	1	11.14
	405	12	70	29	69	1	2	1.24	9.51
	406	12	70	29	69	1	2	1.34	8.68
	407	12	70	29	69	1	2	1.52	8.02
	408	12	70	29	69	1	2	2.8	4.7
	409	12	70	29	69	1	2	3.2	6.95
	410	12	70	29	69	1	2	3.92	5.17
[59]	411	18	80	20.7	91	1	1	0.27	11.05
	412	18	80	20.7	91	1	1	0.33	11.49
	413	18	80	20.7	91	1	1	3.95	12.87
	414	18	80	20.7	91	1	1	3.76	13.2
	415	18	80	20.7	91	1	1	7.17	10.89
	416	18	80	20.7	91	1	1	7.45	12.15
	417	18	80	20.7	91	1	1	10.78	10.34
	418	18	80	20.7	91	1	1	10.45	10.5
	419	18	80	20.7	91	1	1	13.18	7.37
	420	18	80	20.7	91	1	1	13.56	7.92
	421	18	80	44.4	91	1	1	0.3	14.86
	422	18	80	44.4	91	1	1	0.18	16.57
	423	18	80	44.4	91	1	1	3.78	19.4
	424	18	80	44.4	91	1	1	4.13	22.49
	425	18	80	44.4	91	1	1	7.74	19.35
	426	18	80	44.4	91	1	1	7.32	18.58
	427	18	80	44.4	91	1	1	11.52	15.45
	428	18	80	44.4	91	1	1	11.87	14.13
	429	18	80	44.4	91	1	1	14.81	14.08
